# Treatment of stroke in aged male and female rats with Vepoloxamer and tPA reduces neurovascular damage

**DOI:** 10.3389/fneur.2023.1282736

**Published:** 2023-10-06

**Authors:** Li Zhang, Hao Luo, Chao Li, Hua Teng, Brianna Powell, Mei Lu, Michael Chopp, Zheng Gang Zhang

**Affiliations:** ^1^Department of Neurology, Henry Ford Hospital, Detroit, MI, United States; ^2^Department of Biostatistics and Research Epidemiology, Henry Ford Hospital, Detroit, MI, United States; ^3^Department of Physics, Oakland University, Rochester, MI, United States

**Keywords:** stroke, thrombolysis, reperfusion, rats, aging

## Abstract

Stroke is a leading cause of death and disability worldwide, mainly affecting the elderly. Unfortunately, current treatments for acute ischemic stroke warrant improvement. To date, tissue plasminogen activator (tPA) is of limited use in stroke patients mainly due to its narrow therapeutic window and potential for hemorrhagic complication. The adjuvant treatment with Vepoloxamer, a purified amphipathic polymer has been shown to enhance the thrombolytic efficacy of tPA treatment in young adult male rats after embolic stroke. However, most stroke patients are aged; therefore, the current study investigated the therapeutic effect of the combined tPA and Vepoloxamer treatment in aged male and female rats subjected to embolic stroke.

**Methods:**

Male and female Wistar rats at 18 months of age were subjected to embolic middle cerebral artery occlusion and treated either with monotherapy of tPA or Vepoloxamer, a combination of these two agents, or saline at 4 h after stroke onset. Neurological outcomes were evaluated with a battery of behavioral tests including adhesive removal, foot-fault, and modified neurological severity score tests at 1 and 7 days after stroke onset, followed by histopathological analysis of infarct volume. Residual clot size and vascular patency and integrity were analyzed.

**Results:**

The combination treatment with Vepoloxamer and tPA significantly reduced infarct volume and neurological deficits in male and female rats compared to rats treated with saline and the monotherapies of tPA and Vepoloxamer. While Vepoloxamer monotherapy moderately reduced neurological deficits, monotherapies with tPA and Vepoloxamer failed to reduce infarct volume compared to saline treatment. Furthermore, the combination treatment with tPA and Vepoloxamer accelerated thrombolysis, reduced ischemia and tPA-potentiated microvascular disruption, and concomitantly improved cerebrovascular integrity and perfusion in the male ischemic rats.

**Conclusion:**

Combination treatment with tPA and Vepoloxamer at 4 h after stroke onset effectively reduces ischemic neurovascular damage by accelerating thrombolysis and reducing ischemia and tPA potentiated side effects in the aged rats. This funding suggests that the combination treatment with tPA and Vepoloxamer represents a promising strategy to potentially apply to the general population of stroke patients.

## Introduction

Stroke is a leading cause of death and disability, mainly affecting the elderly. Currently, nearly 7.6 million people suffer an ischemic stroke globally each year and the numbers are expected to increase substantially with the aging population (1–3). The present standard of care for acute ischemic stroke is restricted to reperfusion interventions including endovascular thrombectomy and intravenous thrombolysis, primarily with tissue plasminogen activator (tPA), which mitigate stroke-related disability and mortality in a significant proportion of selected patients (4, 5). However, the current reperfusion interventions are limited by infrequent and delayed recanalization, and the risk of hemorrhagic complication (6–11). Moreover, as an FDA approved thrombolytic agent for acute ischemic stroke, the widespread use of tPA is limited largely due to its narrow therapeutic window (12–14). Nevertheless, while rapid recanalization of occluded cerebral vessels with timely thrombolysis and endovascular treatment is clearly beneficial to stroke outcome, futile reperfusion remains a major hurdle for the reperfusion therapies (6–11). Therefore, to improve the window of efficacy and safety of reperfusion interventions, it is imperative to develop novel and complementary agents to accelerate recanalization, while concomitantly protecting the cerebrovascular integrity.

Clinical evidence on thrombi retrieved from stroke patients revealed that the accumulation of platelets and neutrophils within thrombi alters the fibrin network and forms Neutrophil Extracellular Traps (NETs), which render thrombi less susceptible to thrombolysis (15, 16). In addition, we and others have observed a rapid recruitment of blood elements including platelets, fibrin, and neutrophils at the occlusion site and in downstream microvessels that lead to aggravation of perfusion deficits and blood brain barrier (BBB) disruption in experimental models of stroke (17–19). Thus, clinical and experimental data suggest that stroke induced prothrombotic and proinflammatory events on the cerebrovasculature may contribute to thrombolysis resistance and disruption of cerebrovascular integrity (16, 20).

Vepoloxamer is a purified amphipathic polymer and exerts potent anti-thrombotic, anti-inflammatory, and hemorheological properties in experimental neurovascular injury models including stroke and traumatic brain injury (21–23). We have shown that Vepoloxamer treatment reduces microvascular thrombosis *via* blocking stroke provoked platelet aggregation and activation in adult rats and that Vepoloxamer extends the tPA therapeutic window by enhancing thrombolysis and reducing tPA induced cerebrovascular disruption (21). Advanced age exacerbates BBB disruption and accelerates ischemic infarct progression (24–27). Also, individual elderly female and male stroke patients often show different responses to pharmacological inventions (28, 29). Accordingly, we investigated the therapeutic effect of the combined Vepoloxamer and tPA treatment on ischemic neurovascular damage in aged male and female rats.

## Materials and methods

All animal procedures were approved by the Henry Ford Hospital Institutional Animal Care and Use Committee (IACUC). All outcome assessments were performed by observers who were blinded to the experimental groups.

### Embolic middle cerebral artery occlusion (eMCAO) model

Male and female Wistar rats (Charles River Laboratories) at 18 months of age were subjected to eMCAO, as previously described (17, 30). Briefly, for clot preparation, arterial blood (~200 μL) from the femoral artery of a young adult donor rat was collected into a sterile PE-50 tubing. The blood containing PE-50 tubing was then incubated for 2 h at 37°C and stored overnight at 4°C. Before the induction of eMCAO, the clot within PE-50 tubing was flushed out into a saline filled sterile Petri dish and washed with saline for 5 min. A single clot (40 mm for female and 50 mm for male in length) was collected into a saline filled modified PE-50 catheter (0.3–0.4 mm outer diameter at the tip end). The right common carotid artery (CCA), external carotid artery (ECA), and internal carotid artery (ICA) were exposed through a midline incision. The right ECA was isolated and severed between ligations. The proximal stump of the ECA was catheterized with the clot filled modified PE-50 catheter attached to a 100-μL Hamilton syringe. The modified PE-50 catheter was gently advanced into the ICA rostrally for 18–19 mm. The clot was then injected slowly with 5–10 μL of saline. The indwelling catheter was removed immediately after the clot injection and the ECA stump was ligated.

### Treatment groups

Male (*n* = 80) and female (*n* = 40) rats were randomized into 4 groups according to a computer-generated randomization list: 1) saline control, 2) tPA, 3) Vepoloxamer, and 4) Vepoloxamer and tPA. Although tPA at a dose 10 mg/kg is effective to improve stroke outcome in adult rats, the full dose of tPA (10 mg/kg) dramatically increases the mortality rate in aged ischemic rats (31). Thus, recombinant human tPA (Genentech) at a dose of 5 mg/kg was used in the current study. At 4 h after eMCAO, tPA (5 mg/kg) was intravenously injected (IV) at 10% bolus followed by continuous infusion for 30 min via a tail vein cannulation. Vepoloxamer was given intravenously *via* a second tail vein at a bolus dose 300 mg/kg at 4 h after eMCAO, followed by a 12 h continuous infusion of Vepoloxamer (100 mg/kg, IV) starting at 6 h after the first dose. The chosen dose and treatment regimen of Vepoloxamer are well tolerated and have been shown to reduce ischemic brain damage when administered alone or in combination with tPA to adult rats at 4 h after stroke onset (21).

### Neurological tests

A 5-point Longa scale was used to assess the acute neurological status at 1 h after eMCAO (32). Rats with the Longa score between 1 to 4 were enrolled into the experimental groups. Rats with a score of 0 (indicate no neurological impairment) were excluded. Neurological outcome was evaluated by the modified neurological severity score (mNSS), the adhesive removal test, and the foot-fault test at 1 and 7 days after onset of eMCAO.

The mNSS assesses stroke induced motor, sensory, and balance, and reflex impairments on a scale of 0–18 (0, normal score; 18, maximal deficit), as previously described (33).

The adhesive removal test evaluates sensorimotor asymmetries with bilateral tactile stimuli (34). Briefly, 2 pieces of adhesive-paper dots (113mm^2^) were adhered on each forelimb at the distal-radial region of the wrists. Each rat received three trials per testing day. The mean time (seconds, cut-off at 120 s) for the rat to remove the stimulus from the left forelimb was recorded.

The foot-fault test assesses forelimb motor and coordination dysfunction (35). Briefly, rats were allowed to walk freely on an elevated grid surface (85.5 × 25 cm), with grids of different shapes and sizes. The total number of steps made by each forelimb and the number of foot-faults (fall or slip off the grids) for the left forelimb were recorded. Data are presented as a percentage of left foot-faults relative to the total number of steps.

### Histopathological assessment

For measurement of infarct volume, male and female rats (n = 6-8/group) were euthanized 7 days after eMCAO by transcardial perfusion with saline followed by 4% paraformaldehyde. Infarct volume was measured on seven hematoxylin & eosin (H&E) stained coronal sections. Data are presented as the percentage of the ipsilateral indirect lesion volume relative to the volume of contralateral hemisphere, as previously described (17). Intracerebral hemorrhage is defined as blood deposition evident to the unaided eye on the H&E stained coronal sections and is verified by light microscopy examination.

For assessment of residual clot, an additional set of male aged ischemic rats were euthanized at 24 h after eMCAO by transcardial perfusion with saline and followed with 4% paraformaldehyde. The ventral side of brain was imaged with a digital camera. The area of residual clot (mm^2^) within the right intracranial segment of ICA and the origin of the MCA was measured. The brain tissues used for residual clot measurement were processed and embedded in paraffin for immunohistochemistry analysis.

### Immunohistochemistry

Immunofluorescent staining (*n* = 6 rats/group) was performed on brain coronal sections according to our published protocols (36, 37). The following primary antibodies were used: mouse anti endothelial barrier antigen (EBA, 836804, 1:1000, Biolegend), goat anti fibrin/fibrinogen (YBGMFBG, 1:1000, Accurate Chem), rabbit anti-rat thrombocytes (1:4000, Inter-Cell Technologies), and myeloperoxidase (MPO, 1: 500, Dako). For quantification, the numbers of EBA positive vessels with fibrin/fibrinogen, platelet (thrombocyte), and MPO accumulation within the ipsilateral MCA territory were counted and are presented as the average density of immunoreactive vessels relative to the scan area (mm^2^) determined with an MCID image analysis system (Imaging Research). For quantitative analysis of vascular leakage, the numbers of vessels with extravascular fibrin/fibrinogen leakage were counted throughout the ipsilateral MCA territory. Data are presented as the density of vessels with extravascular fibrin/fibrinogen leakage relative to the imaged area.

### Measurements of cerebrovascular perfusion

To examine brain perfusion, fluorescein isothiocyanate (FITC) dextran (2 × 10^6^ molecular weight, Sigma, 1 mL of 50 mg/mL) was administered intravenously to another set of male aged rats 24 h after eMCAO (n = 4/group). Rats were euthanized 30 min after the injection of FITC-dextran. Coronal sections (100 μm, bregma 0.2 to 1.0) within the MCA territory from each rat were imaged with a confocal microscope and the images were analyzed using the ImageJ software (National Institutes of Health, ImageJ1.44c) and FIJI plugin. For quantification of FITC-dextran perfusion, a threshold of intensity, which detects all FITC signals, was applied to each gray scaled image to ensure that the data reflected the original FITC-dextran perfused patterns. Data are presented as the percentage of FITC perfused area within the MCA territory.

### Statistics

Data were evaluated for normality, and ranked data were used for analysis when data were not normally distributed. For the safety evaluation, logistic regression was used to test the treatment effect on early death or incidence of hemorrhage stratified by sex. Repeated measures analysis was performed to study the treatment effect on behavioral outcome over time and for histopathological measurements considering possible treatment interaction as well as treatment by sex interaction. The Global test using generalized estimation equations was used to study the combination treatment effects on the neurological outcome, measured from three behavioral tests at day 7 after stroke (38). The significant treatment interaction detected at value of *p* <0.05 was further evaluated for a super-additive or sub-additive effect, followed by pairwise group comparisons. A significant sub-additive effect indicates that the combination of tPA and Vepoloxamer has a synergistic effect on stroke outcome. All data are presented as mean ± standard error for illustration.

## Results

### Mortality and gross hemorrhage

Mortality and gross hemorrhage rate were evaluated in male and female aged rats allocated for the 7-day behavioral and histopathological evaluations (n = 10/group/sex) as listed in Table 1. There were no statistical differences in mortality rate and the incidence of gross hemorrhage among groups and between sexes. All early deaths occurred within 48 h post eMCAO. These animals had enlarged ipsilateral hemispheric and midline shift, indicating severe brain swelling and damage. Rats that died prior to the study endpoint (7-day) were excluded from further analysis.

**Table 1:** Mortality, gross hemorrhage rate, and study population.

Treatment	Mortality % (number of deaths among total rats)			Gross hemorrhage % (number of rats with gross hemorrhage among total rats)			Number of rats for infarct volume and behavioral evaluation		
	Male	Female	Male + Female	Male	Female	Male + Female	Male	Female	Male + Female
Saline	30 (3 of 10)	30 (3 of 10)	30 (6 of 20)	20 (2 of 10)	20 (2 of 10)	20 (4 of 20)	7	7	14
tPA	30 (3 of 10)	40 (4 of 10)	35 (7 of 20)	40 (4 of 10)	60 (6 of 10)	50 (10 of 20)	7	6	13
Vepoloxamer	30 (3 of 10)	20 (2 of 10)	25 (5 of 20)	30 (3 of 10)	30 (3 of 10)	30 (6 of 20)	7	8	15
tPA + Vepoloxamer	20 (2 of 10)	20 (2 of 10)	20 (4 of 20)	20 (2 of 10)	30 (3 of 10)	25 (5 of 20)	8	8	16

### Vepoloxamer in combination with tPA improves neurological outcome and reduces infarction

Prior to the treatments, ischemic rats had Longa scores that ranged from 1.9 ± 0.6 to 2.1 ± 0.3 at 1 h after eMCAO, which were not statistically significant among experimental groups and between sexes. Thus, the baseline neurological severity was comparable among experimental groups.

**Figure 1:**
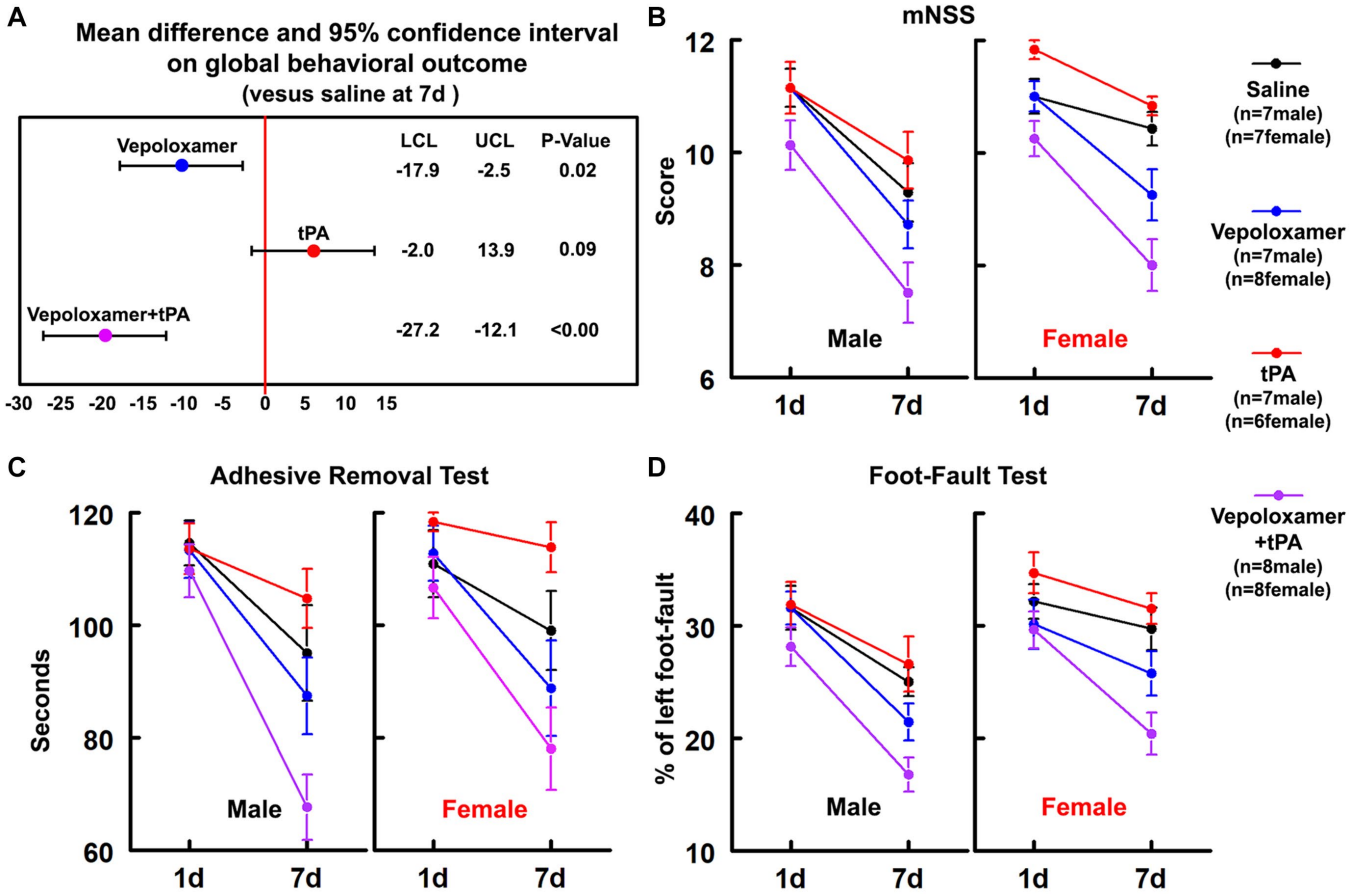
Neurological outcome. **(A)** Shows the global test analysis of overall neurological outcome at 7 days after eMCAO. Treatment of aged male and female rats with Vepoloxamer alone and in combination with tPA significantly reduced neurological deficits at 7 days after eMCAO. The combination treatment with Vepoloxamer and tPA has the maximum effective size. Panels **(B–D)** are the neurological deficits measured by mNSS **(B)**, adhesive removal test **(C)**, and foot-fault test **(D)** in aged male and female rats at 1 and 7 days after eMCAO.

A battery of behavioral tests including mNSS, adhesive-removal test, and foot-fault test were performed at 1 and 7 days after stroke onset to evaluate sensorimotor function. The Global test analysis showed a significant overall treatment effect (*p* < 0.001) on reduction of neurological deficits at day 7 after eMCAO (Figure 1). Compared to saline, tPA alone, or Vepoloxamer alone, tPA in combination with Vepoloxamer significantly (*p* < 0.01) reduced neurological deficits. Compared to saline, the Vepoloxamer monotherapy resulted in moderate, but significant (*p* = 0.02) reduction of neurological deficits, whereas the tPA monotherapy appeared to worsen neurological outcome (*p* = 0.09, Figure 1). Additional analysis based on sex showed that the female rats exhibited significantly worse neurological outcome (*p* = 0.002) than male counterparts across all treatment groups, indicating a sexual dimorphism on stroke outcome in aged rats, which is consistent with clinical findings showing that women with stroke have poorer functional outcomes than male patients (39). However, the effect of the combination treatment on neurological outcome was not sex dependent.

**Figure 2:**
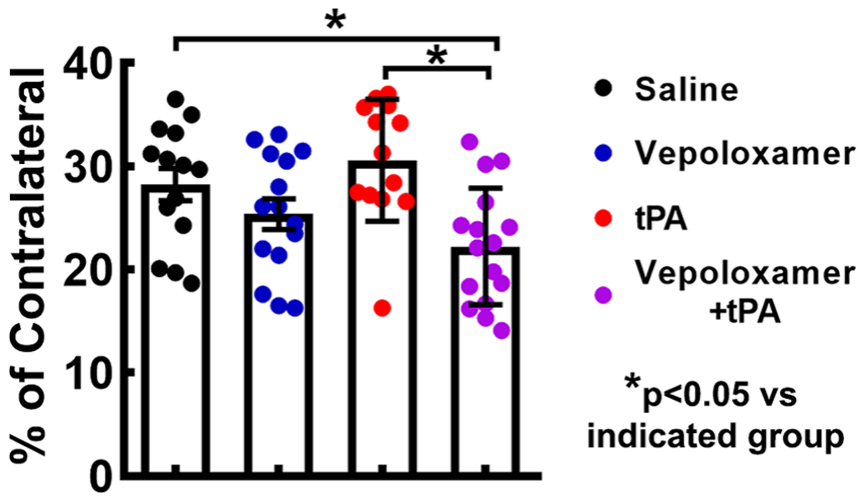
Infarct volume. Bar graph shows the infarct volume in male and female aged rats assessed 7 days after eMCAO.

The combination treatment with tPA and Vepoloxamer significantly reduced infarct volume by 21 and 27% in aged male and female rats compared to saline and tPA monotherapy, respectively. However, monotherapies of tPA and Vepoloxamer failed to reduce infarct volume compared to saline-treated animals (Figure 2). No significant treatment interaction, sex effect, or treatment-by-sex interaction was detected.

### Vepoloxamer in combination with tPA reduces residual clot and thrombosis and increases downstream microvascular perfusion and integrity

**Figure 3:**
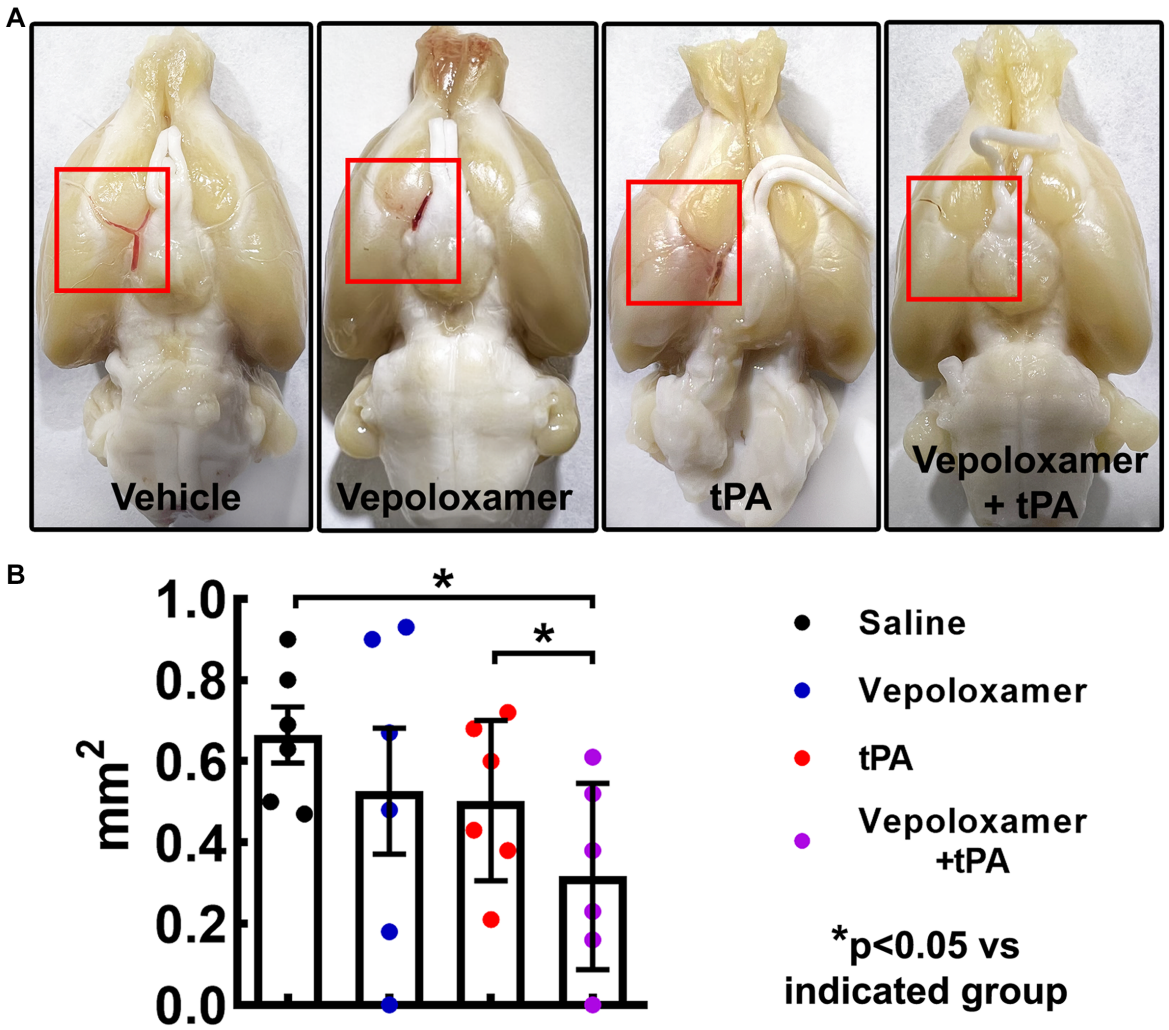
The treatment effects on residual clots. Panels in **(A)** are the representative images of the dorsal surface of rat brains. The boxed areas (red) in **(A)** show the residual clots within the intracranial segment of the right ICA and/or proximal MCA. Quantitative data **(B)** shows the size of residual embolus 24 h after eMCAO.

To examine the treatment effects on thrombolysis, residual clots were measured in the male aged rats 24 h after eMCAO. The residual clot was radially detected within the intracranial segment of the right ICA at proximal MCA in the dorsal surface of well perfused brain (Figure 3). The combination treatment with tPA and Vepoloxamer significantly reduced residual clot size by 60 and 55% compared to rats treated with saline and tPA monotherapy, respectively. However, monotherapies of tPA and Vepoloxamer failed to significantly reduce residual clots compared to the saline treatment (Figure 3).

**Figure 4:**
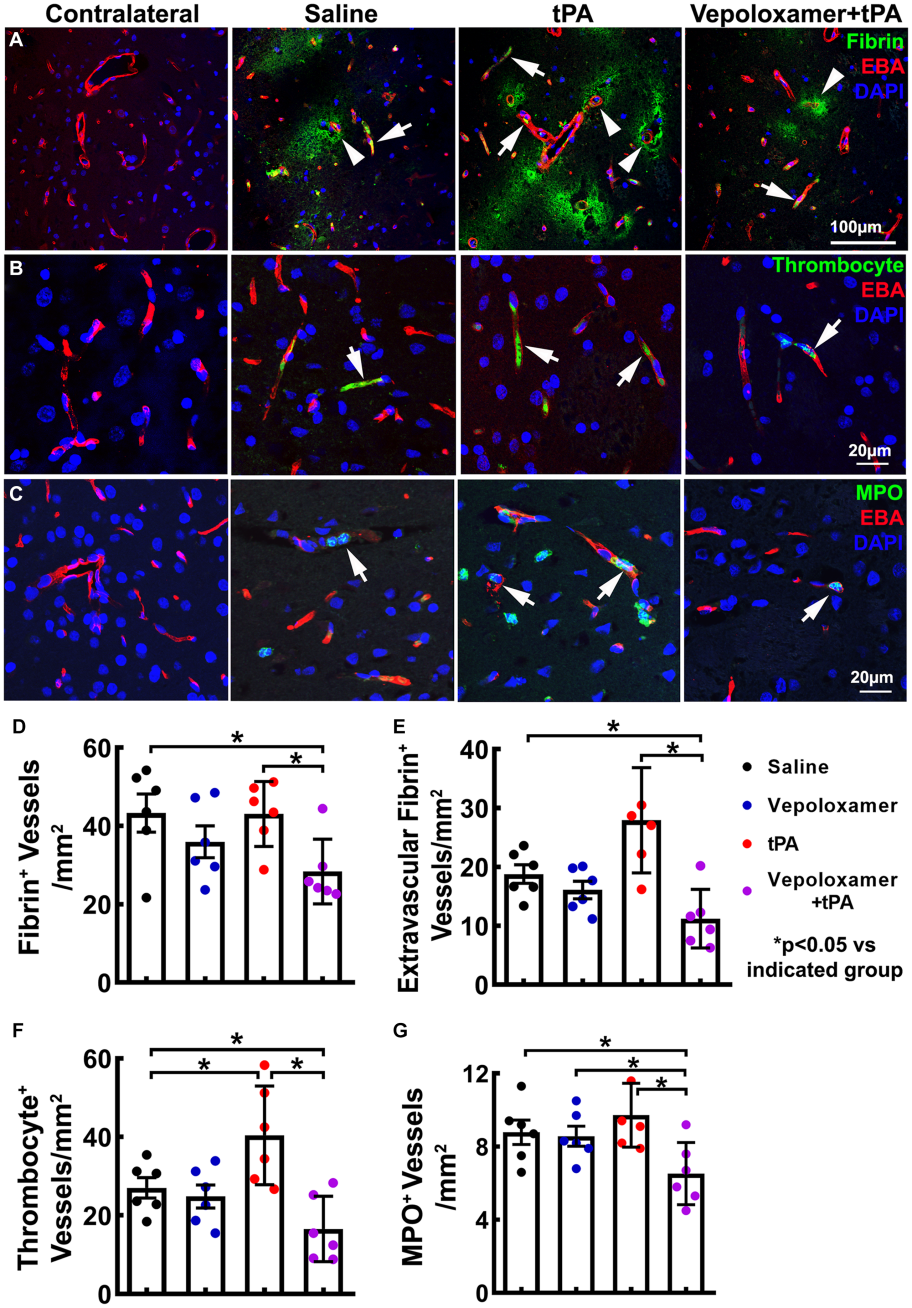
Fibrin/fibrinogen, thrombocyte, and MPO immunoreactivity. Panels in **(A)** are the representative images of double immunofluorescent staining of EBA (red) with fibrin/fibrinogen [**(A)**, green], thrombocyte [**(B)**, green], and MPO [**(C)**, green] obtained from aged male rats treated with saline, tPA alone, and the combination of Vepoloxamer. Intravascular fibrin/fibrinogen, thrombocyte, and MPO immunoreactivity (arrows) and extravasation of fibrin/fibrinogen [arrowhead in **(A)**] are present in the ipsilateral hemisphere. Bar graphs **(D–G)** are the quantitative data of vessels with fibrin/fibrinogen accumulation **(D)**, extravascular fibrin/fibrinogen leakage **(E)**, thrombocyte deposition **(F)**, and MPO immunoreactive neutrophil accumulation **(G)** measured 24 h after eMCAO.

Ischemic stroke provokes downstream microvascular secondary thrombosis characterized by intravascular fibrin/fibrinogen deposition and the recruitment blood cells including platelets and neutrophils, which contribute to the expansion of infarction (18, 19, 40, 41). We then examined the effect of the treatments on downstream microvascular thrombosis, by measuring intravascular fibrin/fibrinogen, platelet, and neutrophil deposition 24 h after stroke onset (*n* = 6/group). The combination treatment of tPA and Vepoloxamer significantly reduced fibrin immunoreactive vessels as well as vessels with thrombocyte positive platelet and MPO positive neutrophil deposition compared to saline control and tPA monotherapy. In addition, significant treatment interactions were observed for platelet and neutrophil accumulation, indicating that Vepoloxamer acts synergistically with tPA to reduce downstream cerebrovascular secondary thrombosis. Vepoloxamer monotherapy did not significantly reduce vascular fibrin, platelet, and neutrophil accumulation, whereas tPA monotherapy resulted in significant increases of microvessels with platelet and neutrophil accumulation compared to the saline treatment (Figure 4). Collectively, our data suggest that the combination treatment with tPA and Vepoloxamer enhances clot lysis and reduces secondary thrombosis.

**Figure 5:**
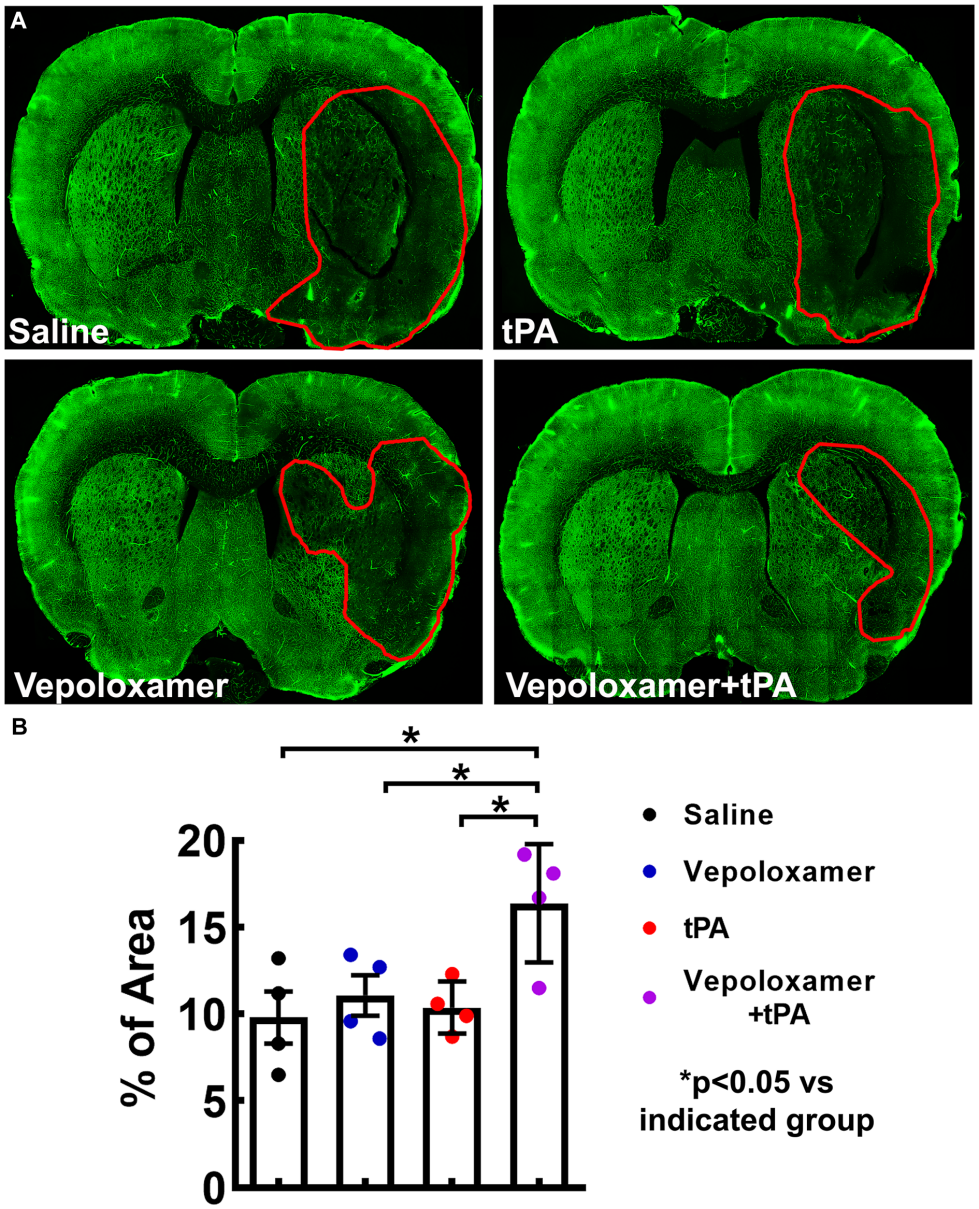
FITC-dextran perfused cerebral vessels. Panels in **(A)** are the representative fluorescent images of coronal sections from rats treated with saline, tPA, Vepoloxamer, and the combination of Vepoloxamer with tPA obtained 24 h after eMCAO. Bar graph **(B)** is the quantitative data showing the percentage of FITC–dextran perfused vessel area within the ipsilateral hemisphere.

Next, we examined cerebral microvascular perfusion by measuring the plasma tracer FITC-dextran perfused vessels within the MCA territory 24 h after stroke onset. The ischemic rats treated with saline exhibited a profound reduction of FITC-dextran perfused area within the ipsilateral MCA territory, indicating perfusion deficit after stroke, whereas the combination treatment of tPA and Vepoloxamer significantly increased FITC-dextran perfusion compared to the ischemic rats treated with saline, tPA alone, and Vepoloxamer alone (Figure 5). A significant treatment interaction (*p* = 0.04) was observed for microvascular perfusion, indicating that the tPA and Vepoloxamer act synergistically to alleviate stroke induced perfusion deficits. Monotherapies of tPA and Vepoloxamer failed to increase FITC-dextran perfusion compared to saline (Figure 5).

We also examined the treatment effects on cerebral microvascular integrity by measuring the extravasation of plasma proteins fibrin/fibrinogen 24 h after stroke onset. The combination treatment with tPA and Vepoloxamer significantly decreased fibrin/fibrinogen extravasation compared to the saline and tPA monotherapy. The beneficial effect of the combination treatment on fibrin/fibrinogen extravasation was synergistic, as indicated by their significant treatment interaction (*p* < 0.01). Monotherapy of tPA significantly increased fibrin/fibrinogen extravasation, while monotherapy of Vepoloxamer did not significantly alter the levels of fibrin/fibrinogen extravasation (Figures 4A,E). Our data indicate that the combination treatment with tPA and Vepoloxamer reduces stroke induced and tPA exacerbated vascular disruption.

## Discussion

Stroke disproportionately occurs among the elderly, with elderly women suffering from increased incidence of stroke and worse stroke outcome (42, 43). With the unmet demand for effective treatments for this large patient population, it is imperative to investigate potential alternative or adjunct therapies in preclinical studies with the consideration of biological variables including age and sex. Here, we provide novel data showing that despite the sex disparity on stroke outcomes in the aged rats, Vepoloxamer acts synergistically with tPA to reduce infarction and neurological deficits in aged male and female rats. The combination treatment with tPA and Vepoloxamer accelerates clot thrombolysis, leading to improvement of downstream cerebral microvascular perfusion and integrity. Thus, Vepoloxamer potentially has translational values to amplify cerebrovascular perfusion and integrity either with tPA and/or endovascular thrombectomy.

We and others have demonstrated that treatment of young adult rats with tPA initiated at 2 h after stroke onset is effective to reduce ischemic brain damage, however, treatment of aged ischemic rats with tPA at 2 h exacerbates neurovascular damage and increases mortality (31, 44). These data are consistent with clinical findings showing that the advanced age is a strong predictor of poor outcome for stroke patients and an important risk factor for intracerebral hemorrhage after thrombolytic treatment (45, 46). Consistently, the present study showed that treatment of aged ischemic rats with the monotherapy of tPA at 4 h after eMCAO had the highest incidence of hemorrhage (50%) among the experimental groups and did not have any therapeutic effects. The incidence of gross hemorrhage in the present study is comparable to studies published by us and others showing an approximately 40% of gross hemorrhage with tPA treatment (47–49). However, Vepoloxamer in combination with tPA significantly reduced infarction and neurological deficits without increasing the incidence of hemorrhage, and the therapeutic effect of the combination was synergistic compared to monotherapies of Vepoloxamer and tPA. These data extend our previous findings of the therapeutic effect Vepoloxamer in the adult ischemic rat by demonstrating that even in the aged ischemic rat Vepoloxamer amplifies the tPA fibrinolytic effect to reduce ischemic neurovascular damage for acute ischemic stroke regardless of animal sex.

We previously demonstrated that a fibrin-rich clot lodged within the MCA rapidly recruits platelets, monocytes and neutrophils to form thrombosis in the model of eMCAO (18). Studies from stroke patient thrombi collected during the endovascular thrombectomy show that thrombi form NETs, which render thrombi resistant tPA-induced fibrinolysis (15, 16). Moreover, delayed tPA administration aggravates neurovascular inflammation, exacerbating cerebral endothelial damage and BBB leakage, which likely mediate the augmentation of cerebral hemorrhage observed in the present study (50–52). Additionally, exosomes released by thrombi and injured cerebral endothelial cells also contribute to impairments of downstream microvascular perfusion and the BBB (53).

Vepoloxamer is a well characterized rheological agent that has been shown to effectively block the hydrophobic adhesive interactions within the blood elements and their attachment to the vascular endothelium, and leads to reduction of blood viscosity (54, 55). In young adult rats, we demonstrated that Vepoloxamer monotherapy reduces residual clots and improves brain perfusion; however, the present study indicates that Vepoloxamer alone does not decrease residual clots and downstream perfusion in aged ischemic rats. It is well established that advanced age is associated impaired blood rheological properties, including increased blood viscosity, enhanced platelet and erythrocyte aggregability, and elevated plasma prothrombotic factors (56–58). Thus, it is likely that the age associated hemorheological alteration hampers the efficacy of Vepoloxamer in the aged rats. Importantly, treatment of aged ischemic rats with Vepoloxamer in combination with tPA robustly enhanced thrombolysis and augmented downstream vascular perfusion and integrity, leading to reduction of neurovascular damage. Our results suggest that Vepoloxamer amplifies tPA fibrinolysis, which overcomes tPA and aging detrimental effects on thrombosis. Clinical studies have shown that timely reperfusion after acute ischemic stroke substantially improves patient neurological outcome (59–61). Thus, the present finding suggests potential clinical value for the use of Vepoloxamer in combination with endovascular thrombectomy to enhance brain perfusion in ischemic stroke patients with large vessel occlusion, because approximately 70% of these patients have incomplete brain tissue perfusion after thrombectomy (62).

In conclusion, our data demonstrated that the combination treatment with Vepoloxamer and tPA at 4 h after stroke onset effectively reduces neurovascular damage in the ischemic aged rats, regardless of sex. Vepoloxamer accelerates thrombolysis and alleviates ischemia and tPA potentiated vascular disruption, and thus represents a potentially safe and effective treatment approach for treatment of stroke patients.

## Data availability statement

The raw data supporting the conclusions of this article will be made available by the authors, without undue reservation.

## Ethics statement

The animal study was approved by Henry Ford Hospital Institutional Animal Care and Use Committee. The study was conducted in accordance with the local legislation and institutional requirements.

## Author contributions

LZ: Conceptualization, Data curation, Funding acquisition, Investigation, Methodology, Project administration, Resources, Software, Supervision, Validation, Visualization, Writing – original draft, Writing – review & editing. HL: Data curation, Investigation, Methodology, Writing – review & editing. CL: Data curation, Investigation, Methodology, Writing – review & editing. HT: Data curation, Investigation, Methodology, Writing – review & editing. BP: Data curation, Investigation, Methodology, Writing – review & editing. ML: Formal analysis, Writing – review & editing. MC: Conceptualization, Supervision, Writing – review & editing. ZZ: Conceptualization, Supervision, Writing – original draft, Writing – review & editing.
